# Supplementary material for: States and traits of neural irregularity in the age-varying human brain
Source: Sci Rep. 2017 Dec 12;7:17381. doi: 10.1038/s41598-017-17766-4 (PMC5727296; doi:10.1038/s41598-017-17766-4)
Supplement: Supplementary file 1 — Supplementary Information [file 41598_2017_17766_MOESM1_ESM.pdf]

Supplementary Information

Supplementary Material

SI Data

Further analysis of behavioural data

Autocorrelation of response patterns. Note that, in principle, our participants could have used a response strategy instead of reporting perceived differences in pitch. Such a strategy, however, would likely result in non-random (i.e., auto-regressive) patterns of the response time series. To address and rule out this potential confound, we analysed the sequence of given responses by computing their autocorrelations and comparing these to autocorrelations resulting from random permutations of participants' response sequences. Precisely, each sequence of responses was permuted 1000 times, resulting in 1000 autocorrelations. Subsequently, for each trial lag, the proportion of correlation coefficients derived from random permutations that exceeded the correlation coefficient of the empirical autocorrelation was calculated. This proportion can be thought of as a p-value, which indicates a significant empirical autocorrelation if $p < .05$. Comparing serial autocorrelations of actual behavioural data with serial autocorrelations of randomly permuted sequences (Fig. S1) did not result in any significant differences ($p > .05$ for all 19 participants and lags). Thus, participant response behaviour did not depend on previous responses or the position of a trial in an experimental block.

Perceptual learning. It is noteworthy that our data fully replicate results from Amitay and colleagues[18,42], which were focused on the perceptual learning resulting from training on such physically identical stimuli: Our participants also showed decreased frequency discrimination thresholds (i.e., were able to distinguish smaller frequency differences) after the experiment, as compared to before (Fig. S2; $t_{18} = 4.5$, $p = .0003$ $r_e = .73$). Since the topic of learning was not of prime importance to the current study and we did not include adequate control conditions, however, we refrain from further interpretation of these behavioural threshold improvements.

Comparison of pre-stimulus and whole trial entropy

We analysed the relation of age and EEG-irregularity by correlating different measures of pre-stimulus and whole-trial entropy with age. As visualized by the almost perfect correlation of average pre-stimulus and whole-trial entropy (r = .99, p = 9.5 x 10^{-22}, Fig. S5), average levels of entropy were comparable between both time windows. Additionally, results in the whole-trial time-window were similar to the ones based on pre-stimulus data. Neither the ICC (r_{ICC} = .58) nor the age-related increase of entropy (r = .73, p = .0004, electrode Cz) paralleled by a decrease in the standard deviation of average entropy across trials (r = -.56, p = .01) appeared to differ substantially. The only systematic difference between the trial-wise averages of pre-stimulus and whole-trial entropy lay in their respective variability across trials with the pre-stimulus measure displaying higher variance overall (t_{18} = 6.8, p = 2.1 x 10^{-6}, r = .85, Fig. S5). This effect obviously stems from the different number of entropy samples that were used to calculate the respective trial mean (pre-stimulus: 16 vs whole trial: 200), resulting in a less variable estimation of average entropy over trials for the whole-trial entropy. All other effects of interest were found to be present and of comparable size in both time-windows, hence we focused on pre-stimulus entropy.

ERP amplitude and oscillatory power do not predict decisions

We compared average evoked responses and oscillatory power between trials of differing decisions using a cluster-based permutation approach[55]. No significant cluster was found when contrasting ERPs of the two different decisions on group level (cluster closest to statistical significance with p = .39, R_e = .58; Fig. 1). Second, no electrode–time–frequency cluster showed a significant power (1-30 Hz) difference between both conditions (Fig. S6; cluster closest to statistical significance with p = .20, R_e = .63).

SI Methods

Pre-experiment adaptive tracking task

Before the main experiment started, an adaptive tracking pitch discrimination procedure was performed to make sure that participants (i) were familiar with the task and (ii) based their decisions on perceived pitch differences only. This becomes necessary when comparing identical stimuli, since fluctuations in the perception of durations and loudness are not only possible but have been reported previously[17]. Before the adaptive tracking task, participants were encouraged to complete 20 practice trials to familiarize themselves with the task. Practice trials consisted of one 650 Hz and one 620 Hz tone, presented in random order (corresponding to the stimuli that were used as a start level of the adaptive tracking paradigm) and involved auditory feedback which was "true" in this case. Subjects were given the chance to ask questions regarding the task before the adaptive tracking paradigm started.

The ensuing adaptive tracking consisted of 30 trials. The same task as for the practice trials was used but pitch differences between the two tones of each pair changed according to an adaptive, one-up–one-down staircase procedure targeting a 50%-correct threshold[61]. After completing the adaptive tracking, subjects were informed via written instructions on the screen that the level of difficulty during the main experiment would be very high (i.e., pitch differences between tones would be small) and that accuracy scores slightly above chance would denote reasonably good performance.

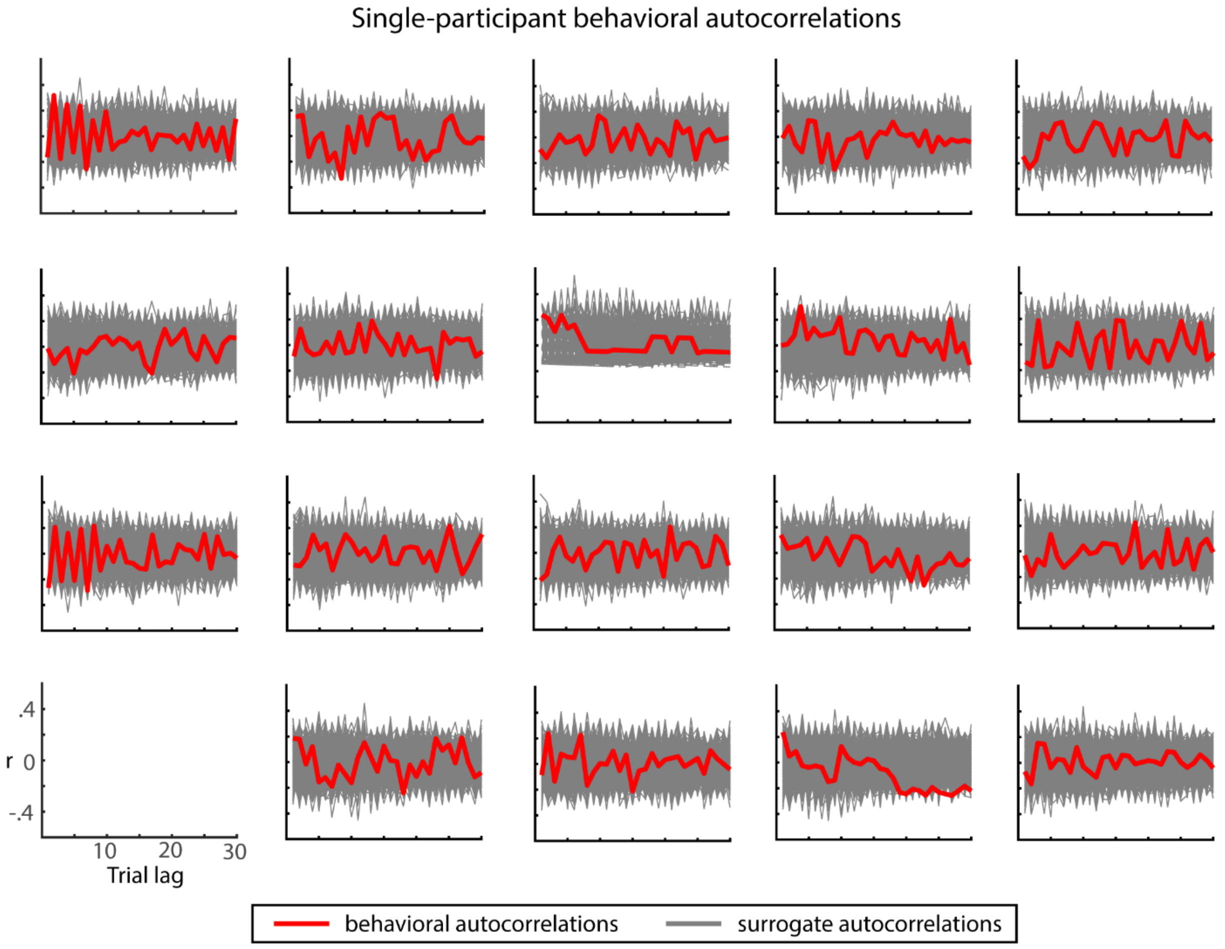

Figure S1. Response sequences of single participants are not substantially autocorrelated. Lagged autocorrelation coefficients (r) of responses are shown for each participant (red lines), for trial lags of 1 to 30. Surrogate autocorrelations (1000 iterations) resulting from permuting every single participant's responses before computing autocorrelations are shown in grey. Note that the observed lagged correlation coefficients of actual responses are well exceeded by surrogate correlations for all participants, indicating no substantial autoregressive structure in the behavioural response patterns.

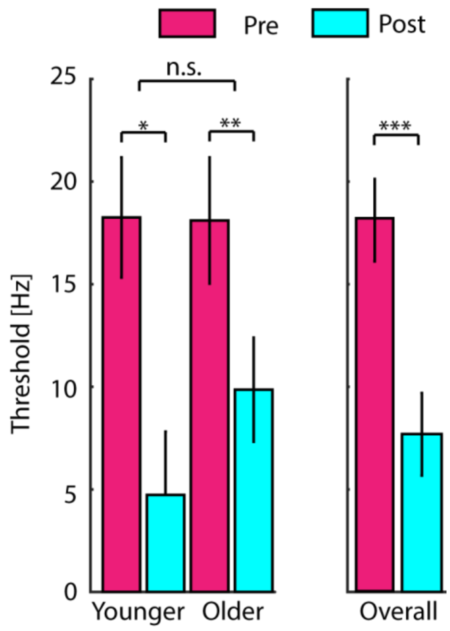

Figure S2. Lowered frequency discrimination thresholds after main experiment (discrimination of physically identical tones). Frequency discrimination thresholds for 50% correct pitch discrimination were assessed using an adaptive tracking procedure before (pink, Pre) and after (cyan, Post) the main experiment. Groups of younger (19–37 years) and older participants (41–74 years) displayed significant decreases in thresholds (left, Post vs. Pre), which did not differ between age groups (and thus obviously were also present over all participants, right). Error bars show ±1 between-subject SEM. * $p < .05$; ** $p < .01$; *** $p < .0005$

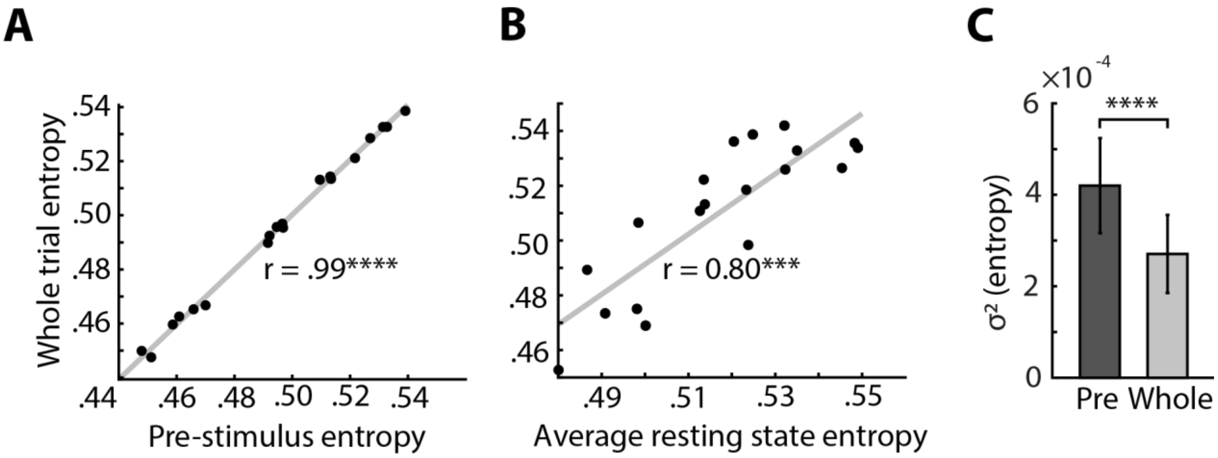

Figure S3. **Comparing EEG-entropy across different time windows and states. A**, EEG-entropy (electrode Cz) averaged across all trials in the pre-stimulus time interval (–2000 to 2000 ms) and average entropy during the whole trial (–.4 to –.1 ms) are highly correlated. Dots represent single participants, least squares line shown in grey. **B**, Similarly, EEG entropy averaged over a resting-state recording and average entropy during task (here, the whole-trial average as also used in **A**) are highly correlated. Dots represent single participants, least squares line shown in grey. **C**, Expectedly, the across-trial variance (σ^2) of pre-stimulus averages (dark grey) is significantly higher than the variance of whole-trial averages (light grey). **** $p < 1 \times 10^{-5}$, *** $p < .0005$

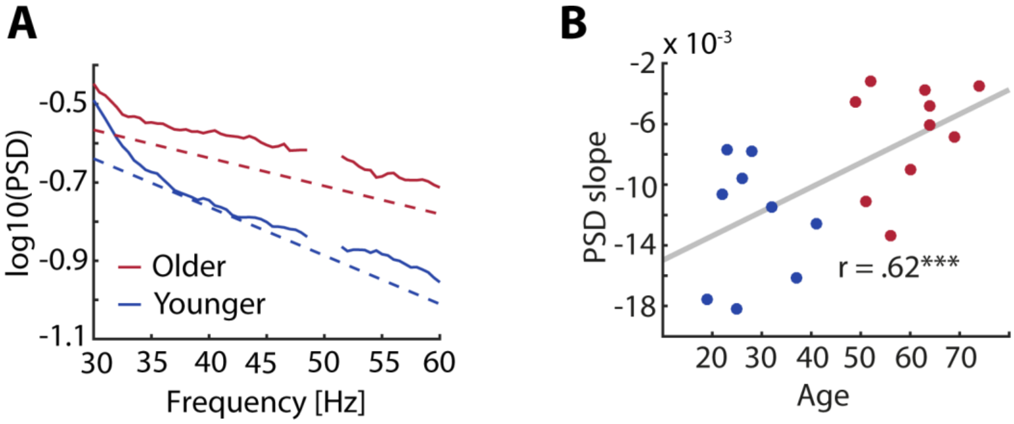

Figure S4. **PSD slopes between 30 and 60 Hz also become more positive with age. A**, Power spectral density (electrode Cz) between 30 and 60 Hz is shown in semi log space for older (red) and younger (blue) participants separately alongside the respective average slope, resulting from the average of linear fits for individual participants across frequencies (dashed lines). Slopes of older participants appear shallower on average. Power between 48.5 and 51.5 Hz represented line noise, thus was excluded for the fit and is also omitted here for visualization. Note that the offsets between linear fits and actual PSD curves are driven by the data of one participant per group, displaying a highly negative intercept. **B**, PSD slopes become less negative (shallower) with increasing age. Again, older subjects are shown in red, younger subjects in blue. *** $p \leq .005$

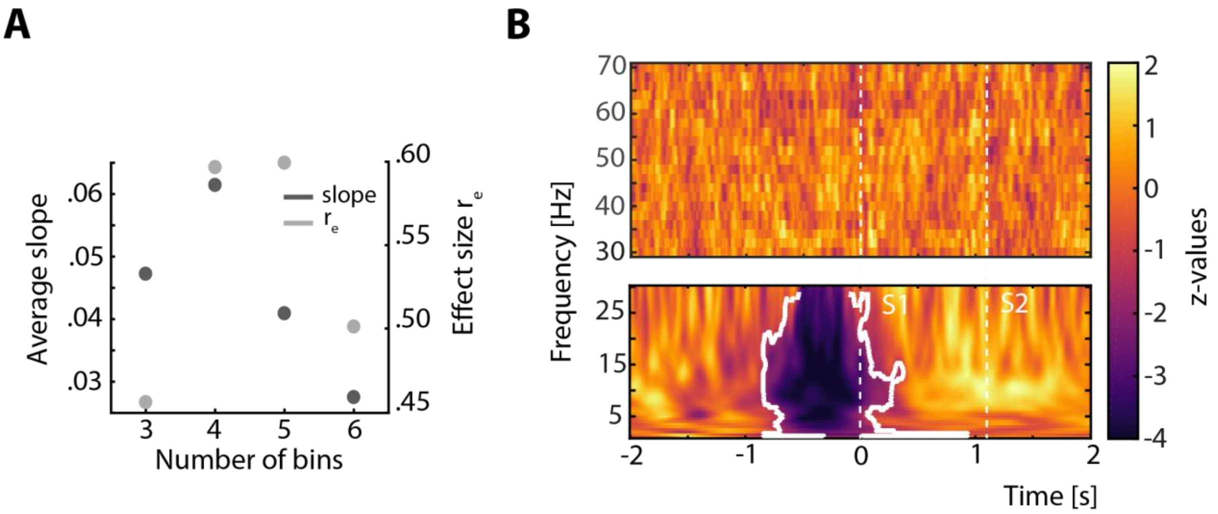

Figure S5. Pre-stimulus entropy impacts behaviour regardless of bin number and tracks low-frequency but not high-frequency power. A, Average slopes fitted across bin-wise probabilities of decisions for S1 are shown (left y axis, dark grey) as a function of the number of used bins (x axis). Corresponding effect sizes r_e are shown in light grey (right y axis). All slopes p<.05. **B**, Z-values from a cluster-based permutation test, modelling the linear increase in oscillatory power across four bins of increasing pre-stimulus entropy, are shown. Note that the bottom panel is replotted from Fig. 3 to allow a comparison to the upper panel (30 - 70 Hz). No significant cluster was found for frequencies higher than 28 Hz.

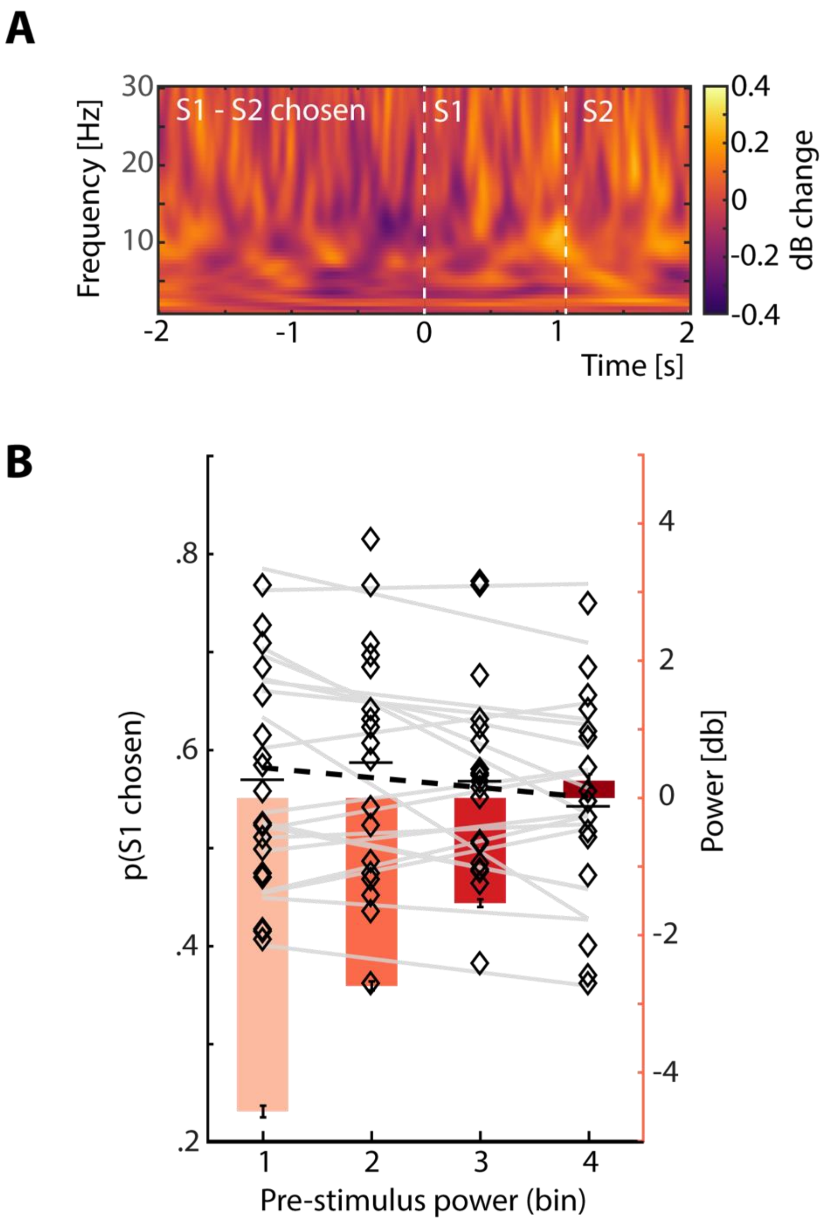

Figure S6. Pre-stimulus power does not differ between decisions. A, Difference in grand average power (dB change, baseline between –2 and –1 s) between trials during which S1 (left) and trials during which S2 (right) was chosen. No significant cluster found (all p >.05). **B,** Probability of choosing S1 is unaffected by pre-stimulus low frequency power. The probability of choosing S1 is shown for each subject and power bin (diamonds). Grey lines depict single-subject slopes fitted to probabilities across bins for visualization. Slopes were not different from zero on average (black dashed line, $p = .1$). Bars of darkening orange represent pre-stimulus low frequency power (± 1 SEM).

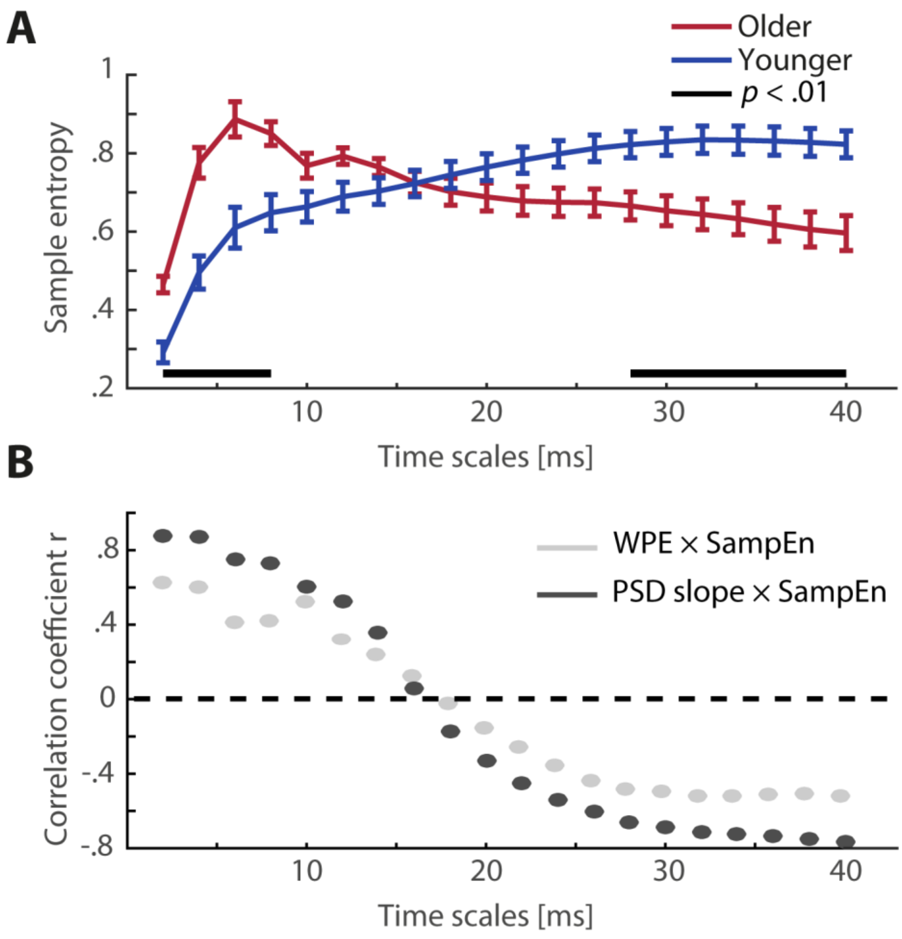

Figure S7. **Multiscale entropy of different age groups and its relation to WPE and PSD slope. A**, Sample entropy (SampEn, ±SEM) at different time-scales (x-axis) averaged for younger (blue) and older (red) participants (electrode Cz) is shown. Note that sample entropy at different time-scales is referred to as multi-scale entropy (MSE). Older participants exhibit higher entropy at finer time scales, a pattern that reverses at coarser time scales (see McIntosh et al.[25]; black bars $p< .01$. **B**, Sample entropy is correlated with average WPE and PSD slope. Whereas this correlation is positive for finer time scales, it switches its sign and is negative for coarser time scales (compare to **A**). The found increase of average WPE and the increased shallowness of PSD slopes with age (compare Fig. 3) thus likely captures processes at finer time scales.
